# Supplementary material for: High-resolution profile of neoantigen-specific TCR activation links moderate stimulation to increased resilience of engineered TCR-T cells
Source: Nat Commun. 2024 Dec 3;15:10520. doi: 10.1038/s41467-024-53911-0 (PMC11615276; doi:10.1038/s41467-024-53911-0)
Supplement: Supplementary file 1 — Supplementary Information [file 41467_2024_53911_MOESM1_ESM.pdf]

Supplementary information

High-resolution profile of neoantigen-specific TCR activation links moderate stimulation to increased resilience of engineered TCR-T cells

Franziska Füchsl*, Johannes Untch*, Vladyslav Kavaka, Gabriela Zuleger, Sarah Braun, Antonia Schwanzer, Sebastian Jarosch, Carolin Vogelsang, Niklas de Andrade Krätzig, Dario Gosmann, Rupert Öllinger, Piero Giansanti, Michael Hiltensperger, Roland Rad, Dirk H. Busch, Eduardo Beltrán, Eva Bräunlein#, Angela M. Krackhardt#

* These authors contributed equally to this work.

# These authors jointly supervised this work.

includes Supplementary Figures 1-16

and Supplementary Tables 1-4

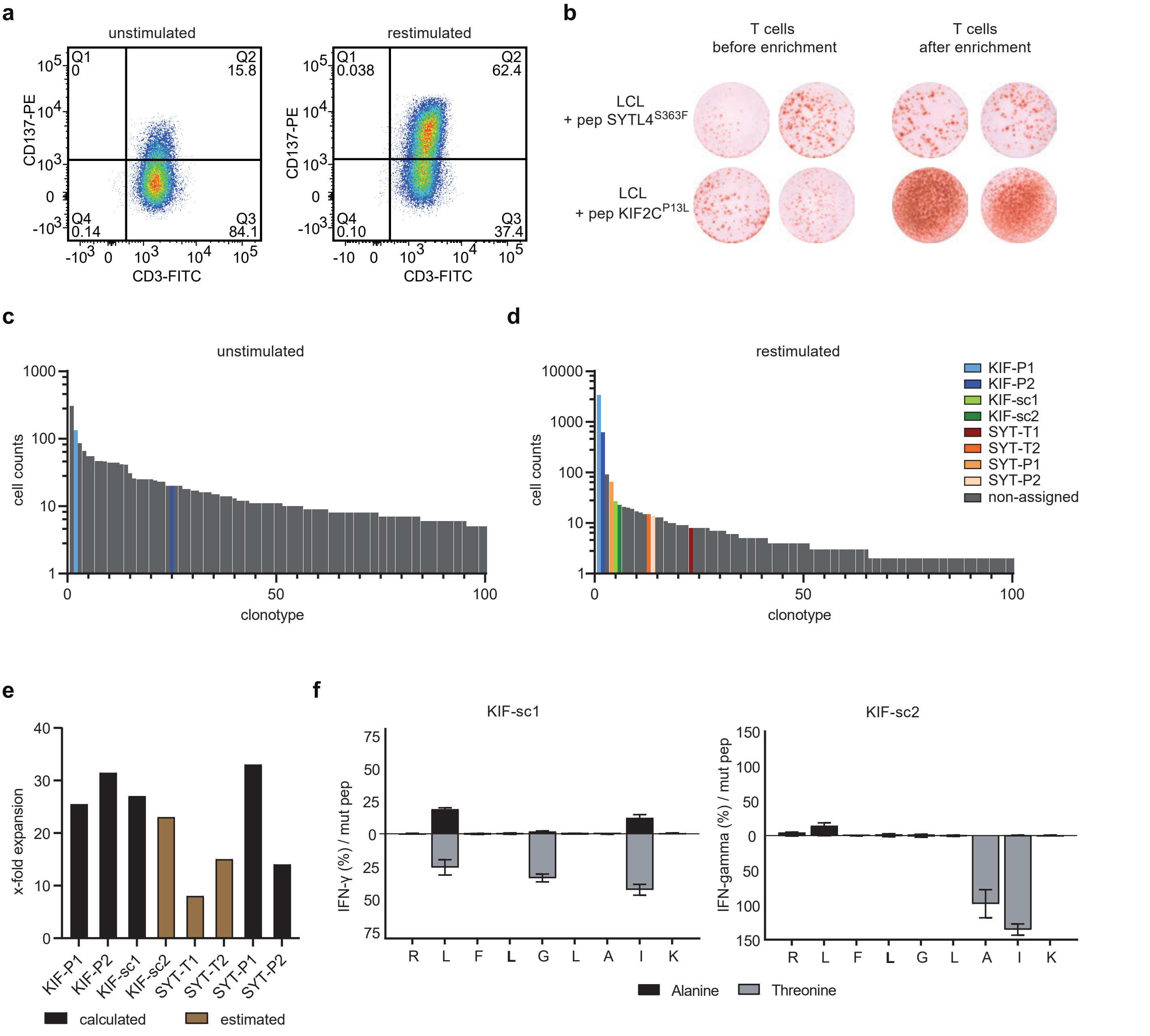

Supplementary Figure 1. CD137-based enrichment of neoepitope-stimulated PBMCs shapes the TCR repertoire and leads to the identification of additional KIF2C^{P13L}-specific TCRs. a, Flow-cytometric assessment of CD137 surface expression measured before and after CD137-specific enrichment of SYTL4^{S363F}- and KIF2C^{P13L}-peptide stimulated PBMCs from patient Mel15. **b**, Comparison of spot forming units (SFU) in IFN-γ ELISpot of Mel15 samples before and after CD137-enrichment. T cells were co-cultured with LCL pulsed with the indicated peptide. **c, d**, Frequencies of the top 100 prevalent TCR clonotypes within the unstimulated (**c**) and stimulated and enriched (**d**) scRNA-seq dataset. **e**, Bar plot showing the fold expansion of neoTCR clonotypes comparing frequency before and after stimulation. For neoTCRs not detected in the unstimulated sample, an estimated ratio was calculated using a theoretical frequency of x=1 read in the unstimulated sample. **f**, Amino acid recognition motif of neoTCRs KIF-sc1 and -sc2 determined by alanine/threonine scan. TCR-tg T cells were co-incubated with target cells pulsed with peptides containing an amino acid substitution with alanine (upper graph) or threonine (lower graph) at the indicated position. Percentage of recognition was calculated as percentage of IFN-γ response in relation to IFN-γ response against target cells pulsed with the original mutated peptide KIF2C^{P13L}.

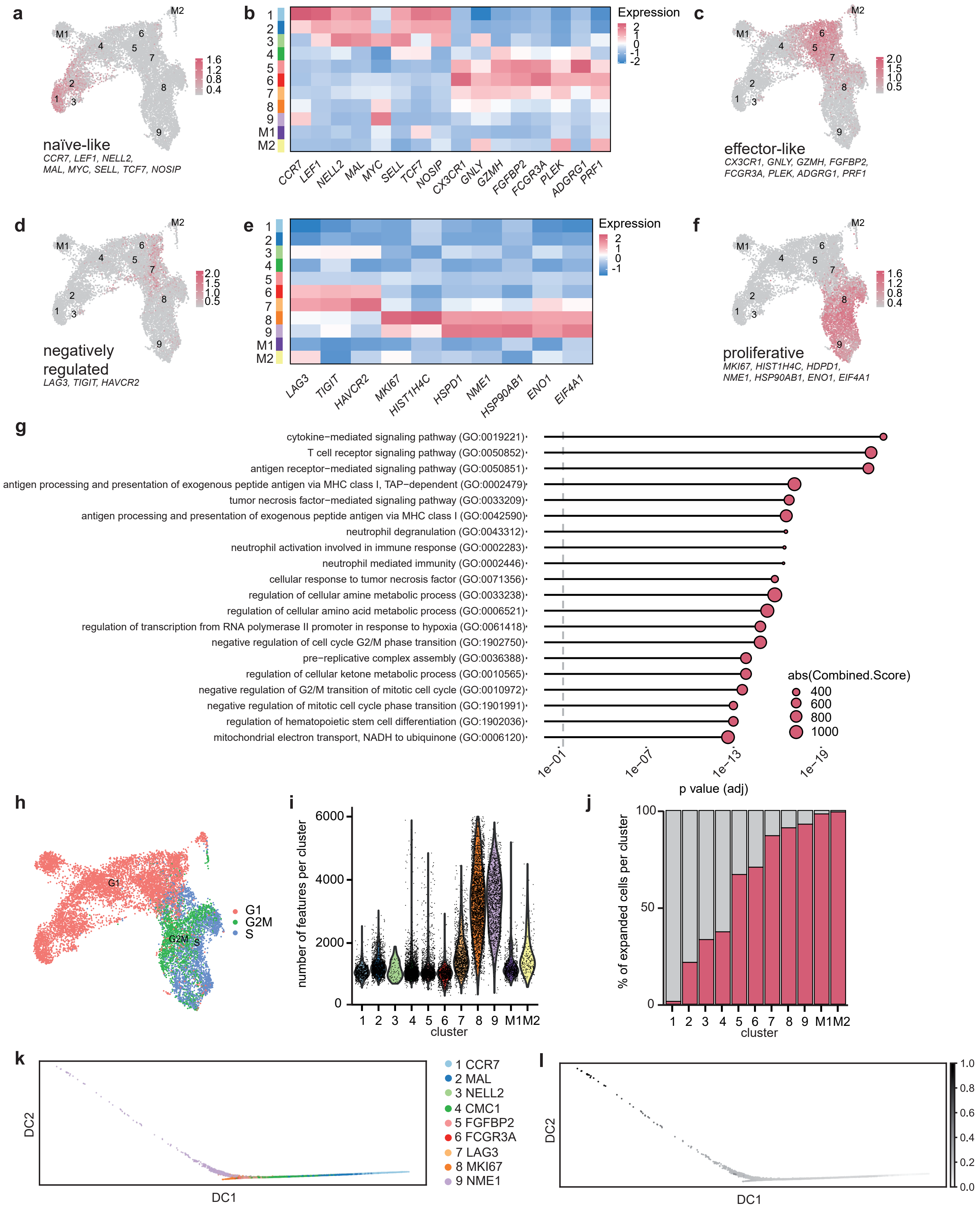

Supplementary Figure 2. Expression profiles of TCR clonotypes were compared as assessed by scTCR-/scRNA-sequencing. a-c, Naïve-like/antigen-inexperienced and effector signature. UMAPs indicating distribution of naïve-like/antigen-inexperienced (**a**) or effector-like (**c**) phenotypes. Heatmap (**b**) showing the scaled average expression of the transcriptomic markers used to generate signature scores as highlighted in **a** and **c**. **d-f**, negatively regulated and proliferative signature. UMAPs showing negatively regulated (**d**) and proliferative (**f**) cells. Heatmap of transcriptomic markers (**e**) integrated into signatures depicted in **d** and **f**. **g**, Top 20 gene ontology biological process (GO:BP) terms of the pathway enrichment analysis of the 7_LAG3 differential gene expression signature. **h**, UMAP indicating transcriptomic analysis of cell cycle phase of each cell. Color code indicates G1 (red), G2M (green) and S (blue) phase. **i**, Violin plot showing number of identified genes per cell within the clusters. **j**, Bar plot indicating percentage of expanded cells within each cluster (clonotypes with two or more cells count as expanded). **k**, Diffusion map using the cells of the 9 clusters excluding MAIT cells. **l**, Pseudotime scores plotted on the diffusion map. Starting point was set on the most naïve cluster (1_CCR7).

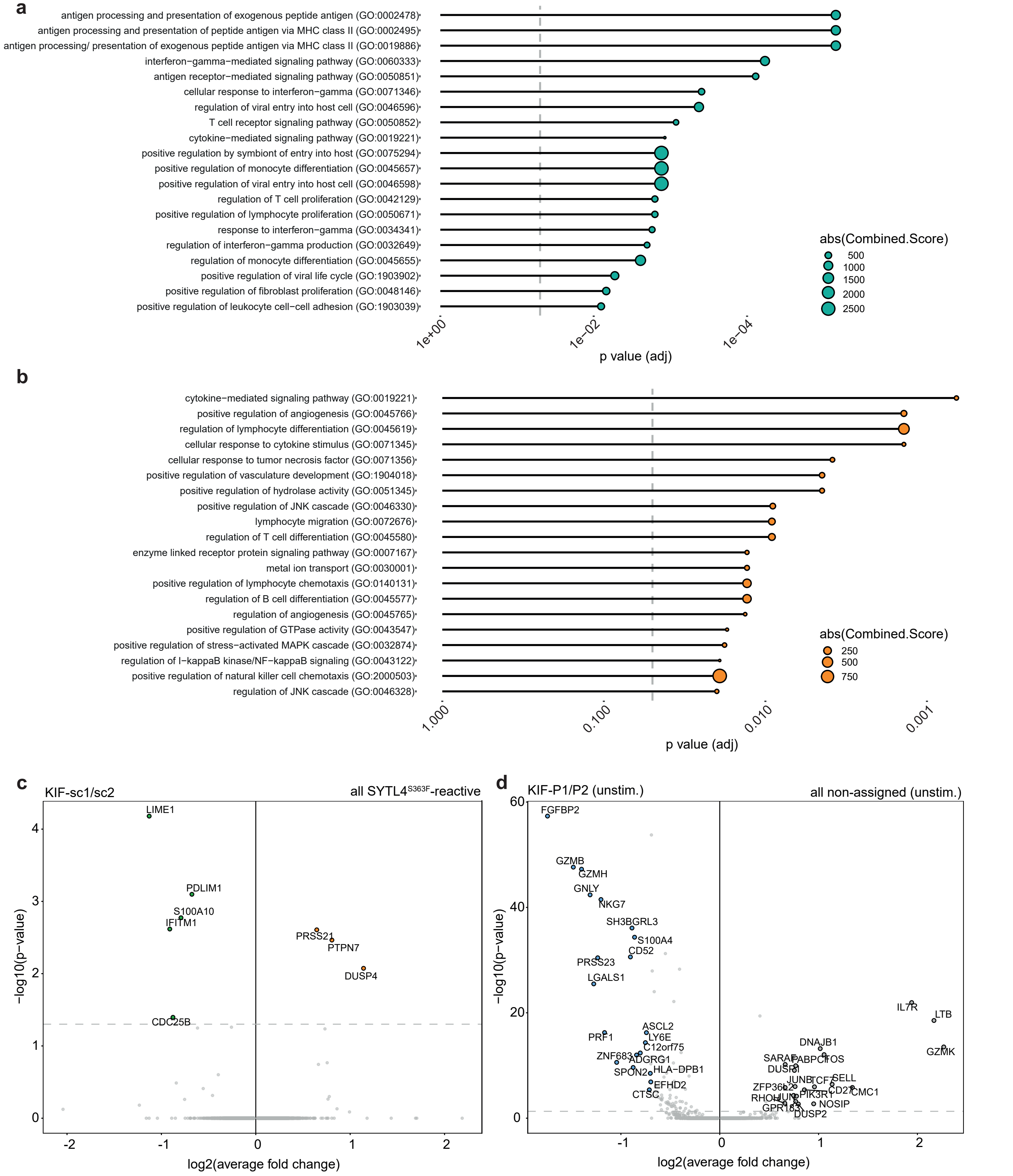

Supplementary Figure 3. Comparison of transcriptomic activation signatures of neoTCR-groups in the repertoire of patient Mel15. **a**, Top 20 GO:BP terms of the pathway enrichment analysis of KIF2C^{P13L}-specific TCRs. **b**, Top 20 GO:BP terms of the pathway enrichment analysis of SYTL4^{S363F}-specific TCRs. **c**, Volcano plot comparing fold changes of transcriptomic expression in all SYTL4^{S363F}-specific TCRs against KIF-sc1/-sc2 TCRs. **d**, Volcano plot showing differential transcriptomic gene expression of unstimulated TCRs KIF-P1 and -P2 in comparison to all non-assigned clonotypes within the unstimulated T cell fraction.

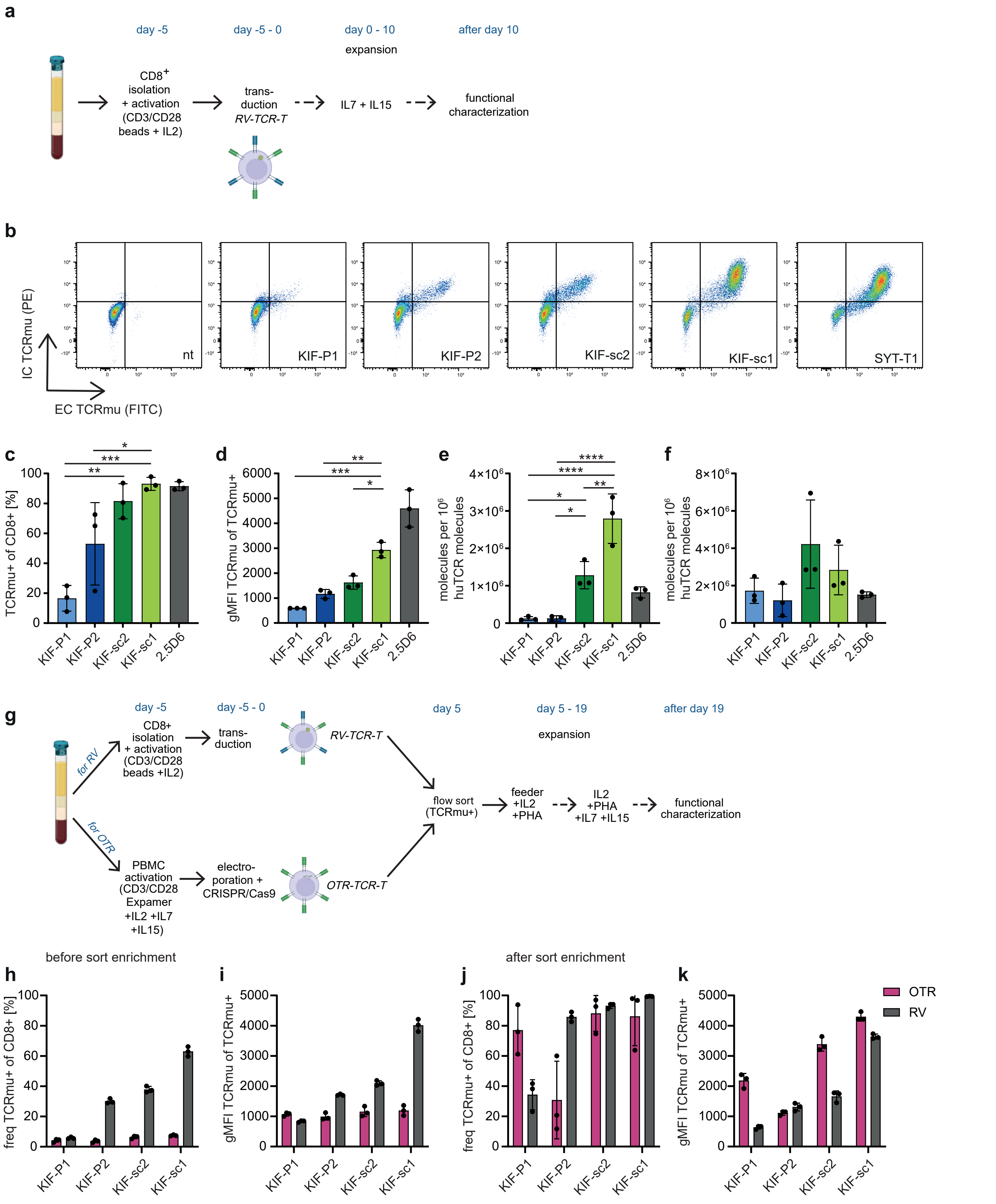

Supplementary Figure 4. Distinct, construct-inherent expression levels of neoTCR constructs detected in different engineering systems. a, Schematic experiment setting of TCR-tg T cell generation in the retroviral engineering (RV) system. Created in BioRender. Bräunlein, E. (2024) *BioRender.com/u76a082*. **b**, Extracellular (EC; FITC) and after permeabilization intracellular (IC; PE) TCRmu-staining on representative TCR-tg T cells from one human donor for different neoTCRs. nt: non-transduced. **c, d**, EC FACS TCRmu-staining on tg $CD8^+$ T cells from three new, different human donors. Frequency of $TCRmu^+$ (**c**) and gMFI of TCRmu on all $CD8^+/TCRmu^+$ (**d**) are depicted. NeoTCRs are compared to irrelevant control 2.5D6. **e**, Number of neoTCR transcripts per endogenous human TCR-β-chain transcript (huTCR) determined by qPCR. **f**, Number of DNA insertions of neoTCR per endogenous human TCR-β-chain (huTCR) determined by qPCR of DNA. For **c-f**, mean and SD of the same three human donors are depicted. **g**, Schematic experiment setting adapted to the orthotopic TCR replacement (OTR) expression systems for comparison of OTR- and RV-engineered T cells. Created in BioRender. Bräunlein, E. (2024) BioRender.com/l53k051. **h-k**, EC FACS staining of TCR expression level depicted by frequency of $TCRmu^+$ cells and gMFI of TCRmu of RV- or OTR-engineered T cells on all $CD8^+/TCRmu^+$ for three biological replicates. gMFI and frequency are depicted before (**h, i**) and after (**j, k**) FACS-based enrichment of TCR-engineered T cells.

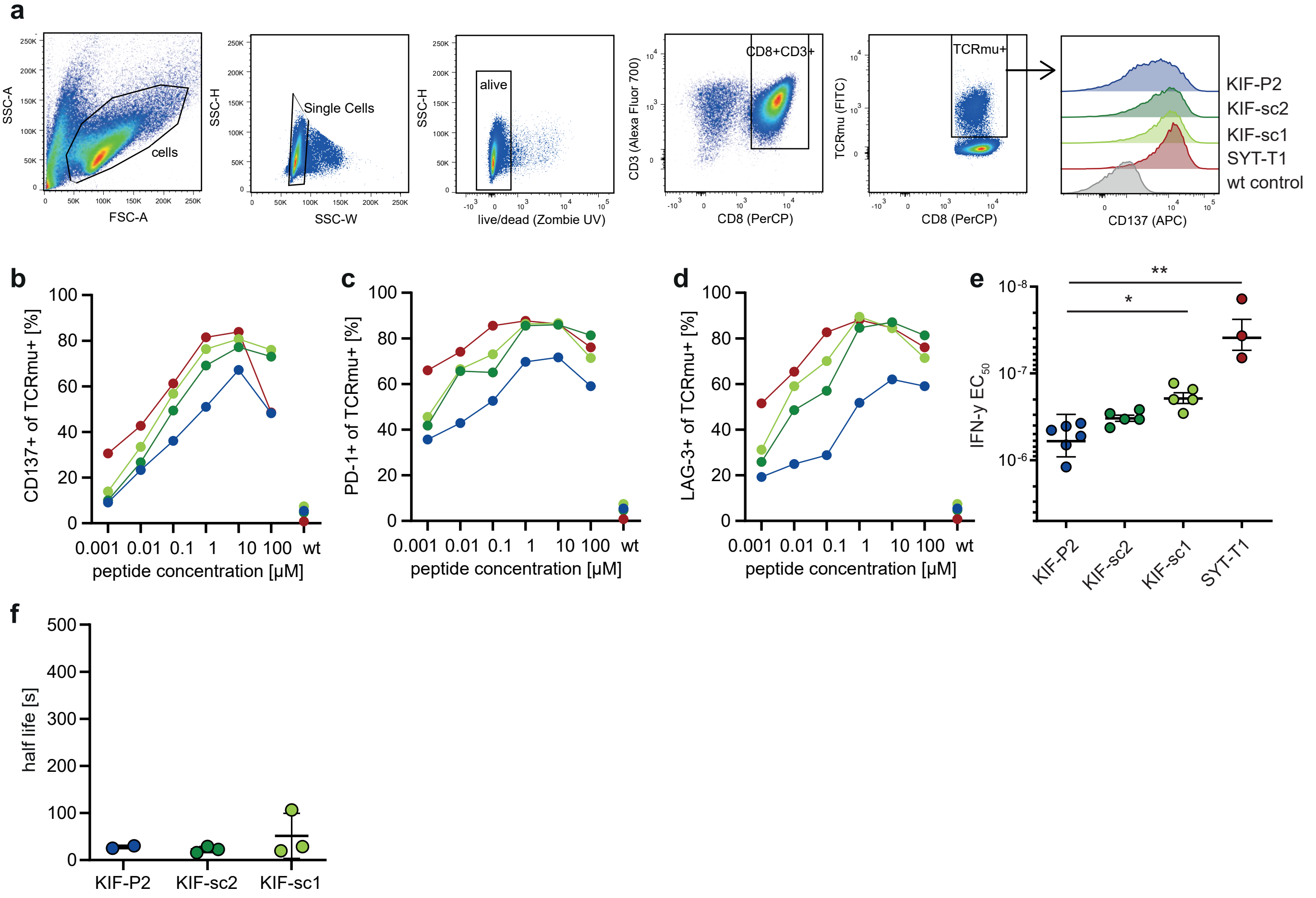

Supplementary Figure 5. Slight differences in functional avidity, but comparable levels of structural avidity detected for KIF-P2, -sc1 and -sc2. a, Exemplary FACS gating strategy for in vitro co-culture assays from left to right. Shown here: Pseudocolor plots of KIF-sc1 (1 µM mut peptide) sample from one donor for peptide titration curve. Plot on the right depicts histograms of TCRmu$^+$ population for CD137 expression on different activated neoTCR-tg T cells compared to a wt-peptide-pulsed control. **b-d**, EC FACS of CD137 (**b**), PD-1 (**c**) and LAG-3 (**d**) for TCR-tg T cells after 24 h of co-culture with Mel15 peptide-pulsed target cells. Corresponding frequencies of CD3$^+$CD8$^+$/TCRmu$^+$ T cells to Figure 3b-c shown here. **e**, Assessment of EC_{50}-values for three KIF2C^{P13L}-reactive neoTCRs and SYT-T1 for comparison of IFN-γ-secretion measured by ELISA from co-cultures with peptide-pulsed T2 cells for several different donors without equalizing TCRmu$^+$-transduction rates. (E:T = 10,000 T cells:10,000 tumor cells). Statistical significance is calculated with one-way ANOVA and Tukey's multiple comparison test (*$p \leq 0.05$, **$p \leq 0.01$). KIF-P2 and SYT-T1 contain pooled results from previously published dataset [31] and novel experiments for direct comparison, as stated in Supplementary Table S4. **f**, FACS-based assessment of k_{off} rates using pMHC-multimers on TCR-tg T cells. No significant differences between k_{off} rates were detected by one-way ANOVA and Tukey's multiple comparison test.

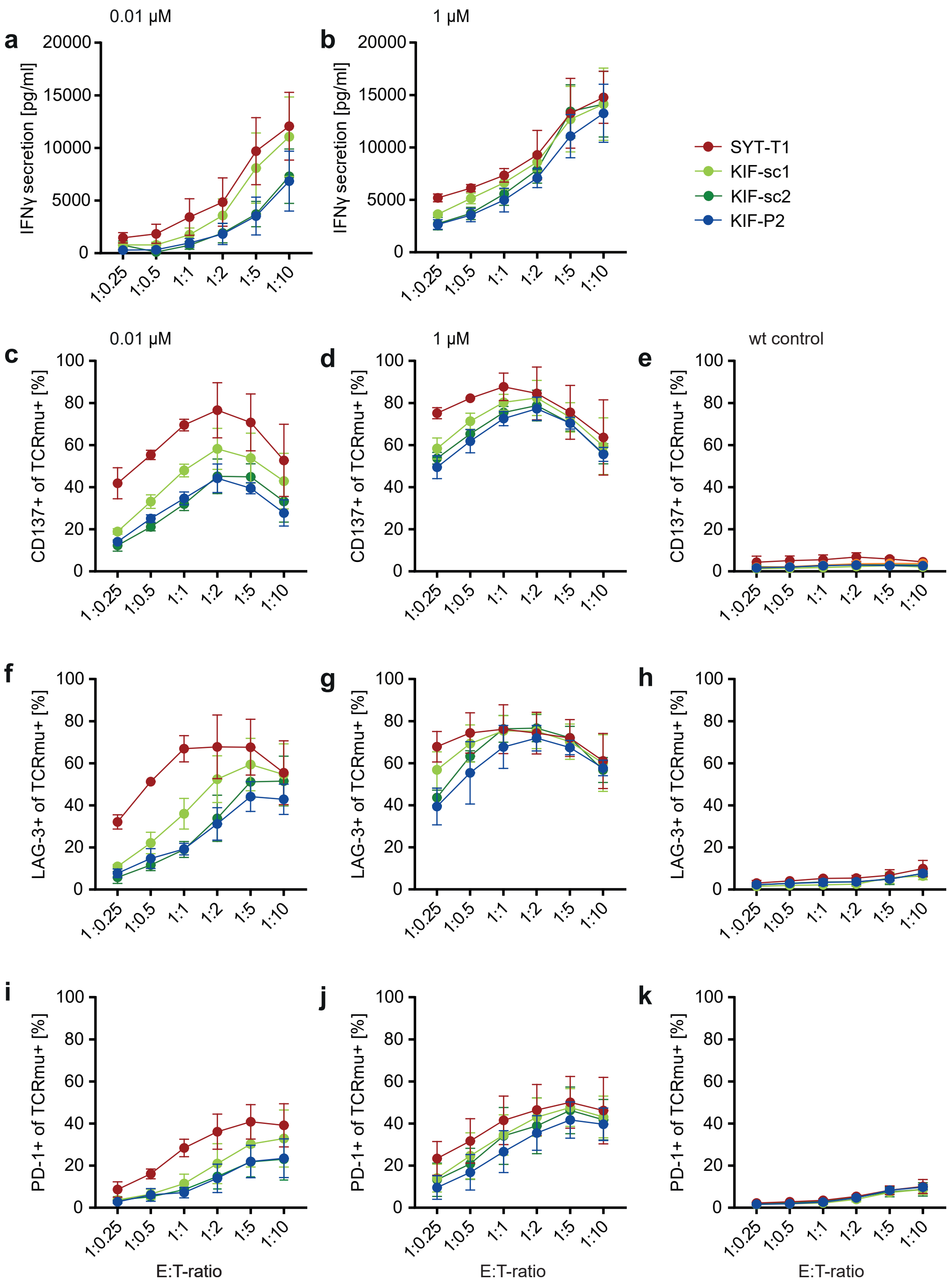

Supplementary Figure 6. Titration of target cell number confirms consistency of activation patterns across different effector-to-target (E:T) ratios. In vitro co-cultures of constant number of TCR-tg T cells (60,000 total T cells, 12,000 TCRmu$^+$ cells) and varying numbers of peptide-pulsed Mel15 LCL as target cells (3,000-120,000) were set up for 20 h and IFN-γ secretion by ELISA (**a, b**), as well as activation markers CD137 (**c-e**), LAG-3 (**f-h**) and PD-1 (**i-k**) by EC FACS analysis, were determined. Two different levels of mut peptide – 0.01 (**a, c, f, i**) and 1 µM (**b, d, g, j**) – were compared. Pulsing with 1 µM wt peptide served as wt-background control (**e, h, k**). Mean and SD of experiments on TCR-tg T cells from three healthy human donors are depicted.

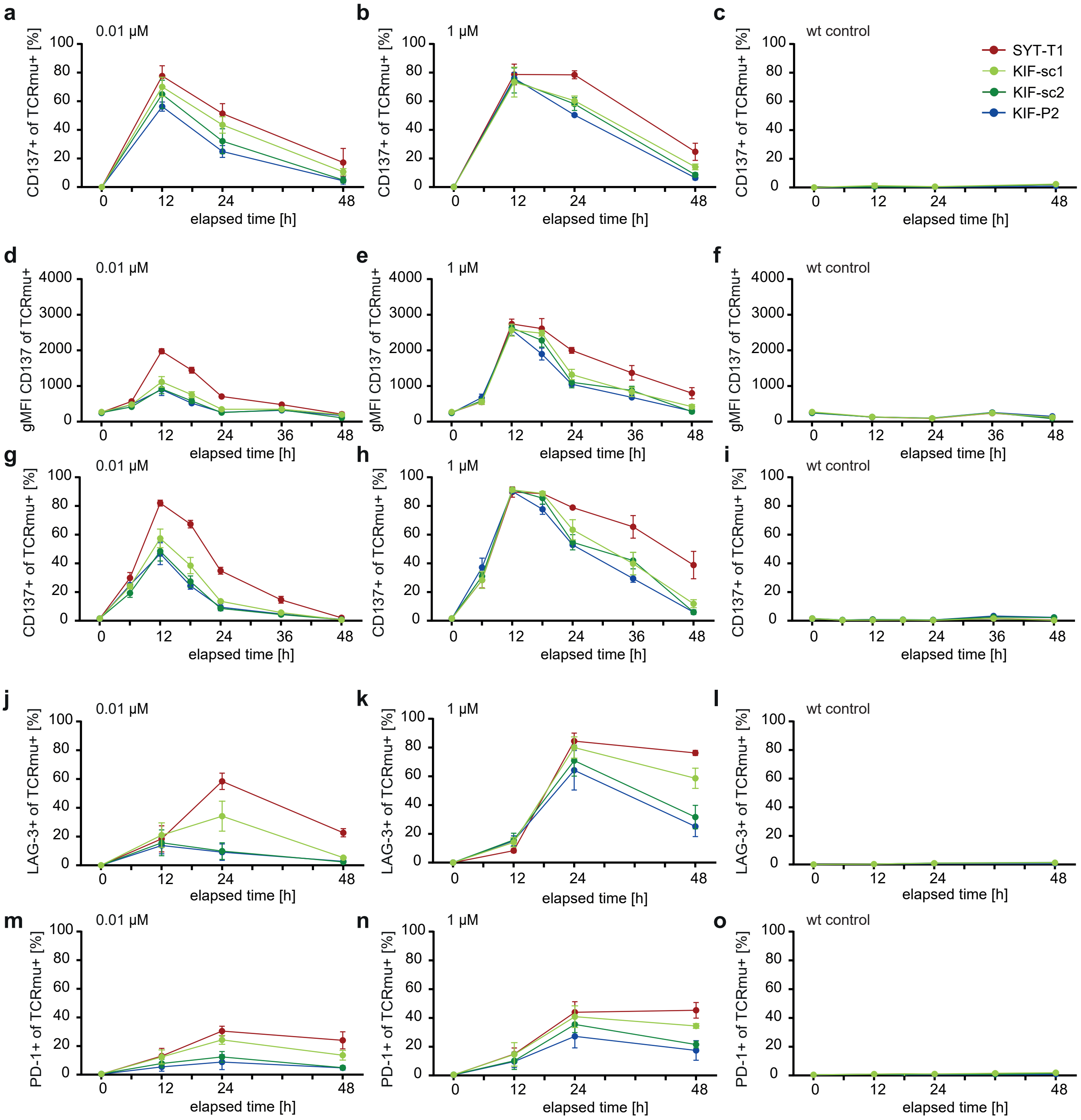

Supplementary Figure 7. Temporary kinetics of surface expression of activation markers reflect strong versus moderate activation patterns of neoTCR-tg T cells. a-c, EC FACS staining of CD137 at different timepoints after co-culture setup with JJN3 B27 peptide-pulsed target cells (E:T = 15,000 tg T cells:15,000 tumor cells). **d-i**, EC FACS staining of CD137 gMFI (**d-f**) and frequency (**g-i**) of TCRmu$^+$ at different timepoints after co-culture setup with peptide-pulsed Mel15 LCL; E:T = 1:1 (10,000 tg T cells:10,000 tumor cells). **j-o**, EC FACS staining of LAG-3 (**j-l**) and PD-1 (**m-o**) at different timepoints after co-culture setup with JJN3 B27 peptide-pulsed target cells in the same experiment as in **a-c**. For all co-cultures a weak (0.01 µM; **a, d, g, j, m**) versus a strong (1 µM; **b, e, h, k, n**) stimulus were compared. Pulsing with 1µM wt peptide served as wt-background control (**c, f, i, l, o**). Frequencies of TCRmu$^+$ T cells in **a-c** and **j-l** are shown correspondingly to Figure **3e-h**. Mean and SD of experiments on TCR-tg T cells from three healthy human donors are depicted.

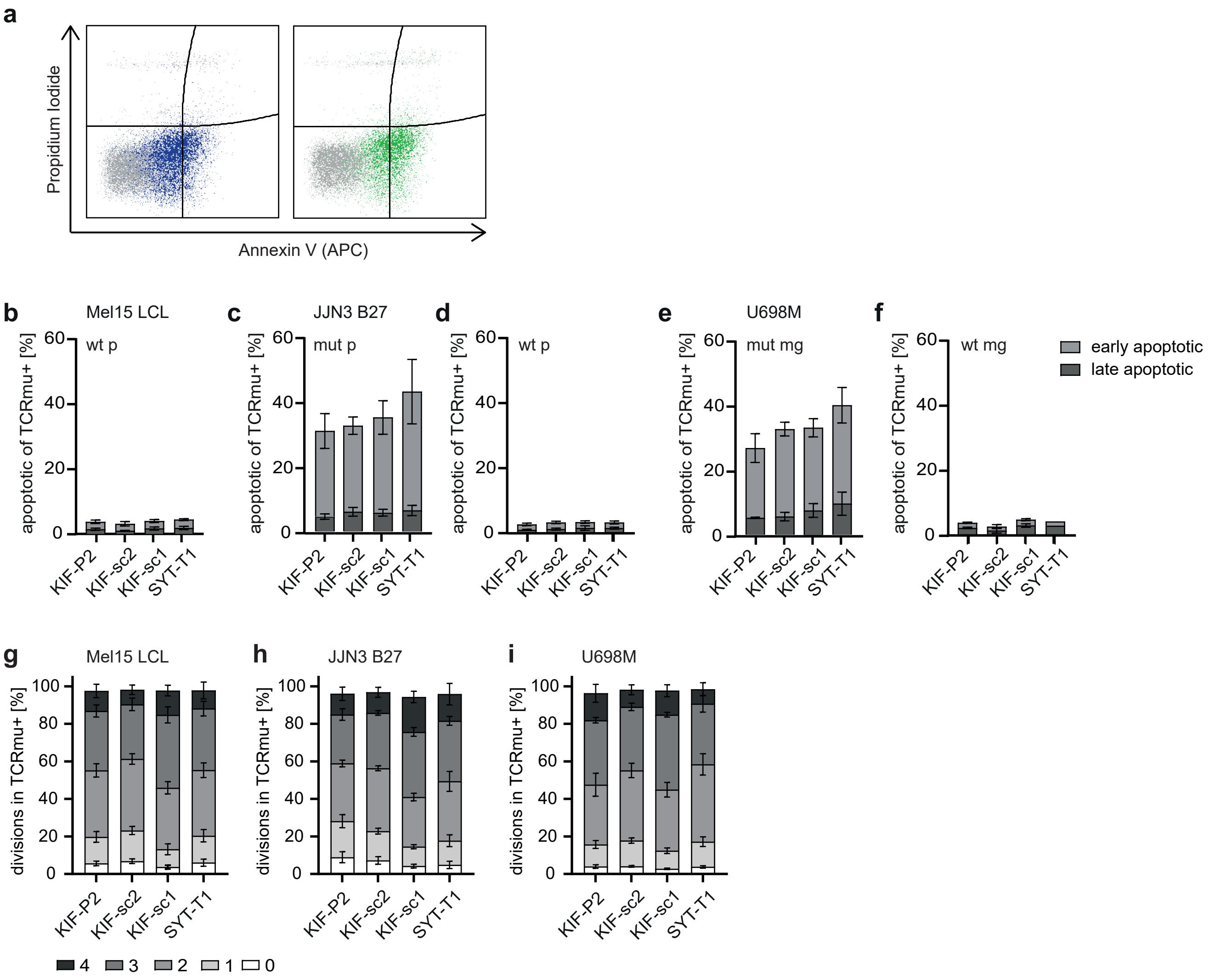

Supplementary Figure 8. Strong activation pattern is associated with higher rates of activation induced cell death (AICD), but no proliferative impairment in vitro. a-f, Annexin-V/PI-staining was employed for detection of activation induced cell death (AICD) upon strong stimulation with 1 µM mut-peptide pulsed target cells after 20 h of co-culture (early apoptotic = Annexin-V$^+$PI$^-$, late apoptotic = Annexin-V$^+$PI$^+$). E:T = 1:1 (15,000 tg T cells:15,000 tumor cells). **a**, exemplary FACS gating for Annexin-V and PI on cells/single cells/CD3$^+$CD8$^+$/TCRmu$^+$ for KIF-P2 (blue) compared to KIF-sc1 (green) of a representative donor. Wt-background is depicted in grey for both plots. **b-f**, Additional data accomplishing Figure 3i (co-culture with Mel15 LCL pulsed with mut peptide), while here a control for Mel15 LCL pulsed with 1µM wt-peptide is depicted (**b**). The same setting was repeated for co-cultures with peptide-pulsed (mut p and wt p) JJN3 B27 (**c, d**) and mut-mg- or wt-mg-tg U698M (**e, f**) tumor cells. **g-i,** CTV-labelling prior to co-culture was used to detect the frequency of cells per number of divisions (0 to 4 divisions) after 4 days of co-culture. Frequencies of divisions of all TCRmu$^+$ cells are shown for co-cultures with Mel15 LCL (**g**), JJN3-B27 (**h**) or U698M-mut mg target cells (**i**). For **b-i** technical triplicates of one donor were pooled prior to staining; mean and SD for biological replicates from three different human donors are shown.

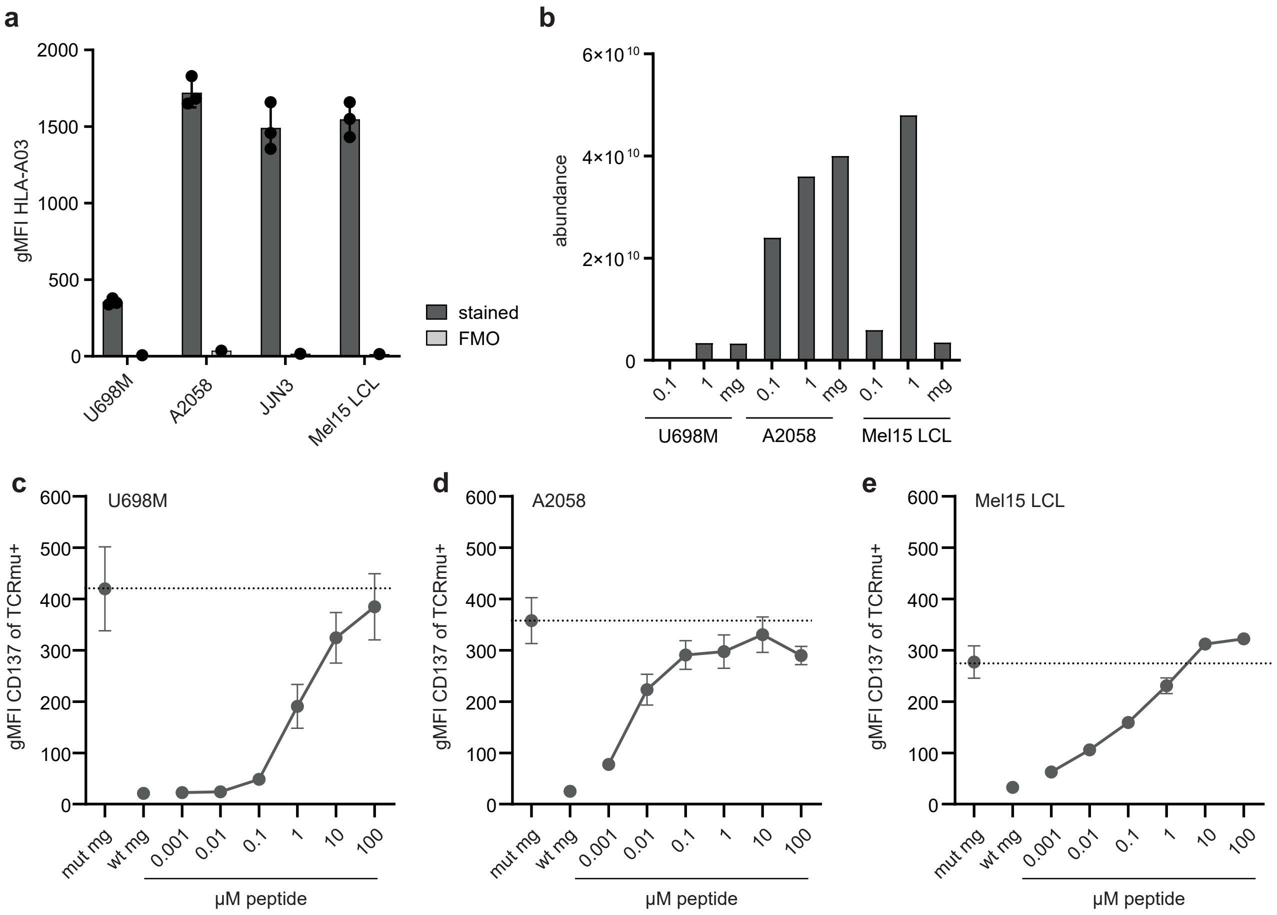

Supplementary Figure 9. Different levels of HLA- and quantitative KIF2C^{P13L} surface expression comparing different cell lines. **a**, EC FACS staining of HLA-A03 was performed in technical triplicates for tumor cell lines U698M mut mg, A2058 mut mg, JJN3 B27 mut mg and Mel15 LCL mut mg. gMFI of the stained sample as well as fluorescence minus one (FMO) control for all tumor cell lines are depicted. **b**, Abundance (in arbitrary units) showing the peak intensity or area under the curve (from the LC-MS trace) of the detected peptide KIF2C^{P13L} upon immunoprecipitation of $1.5x10^8$ tumor cells either after in vitro peptide pulsing with 0.1 or 1 µM peptide (0.1 and 1) or genetic modification via minigene-transduction (mg). One representative sample was measured for each condition, comparing three different cell lines: U698M, A2058 and Mel15 LCL. **c-e**, EC FACS staining of $CD2^+CD8^+$/$TCRmu^+$ T cells for CD137 expression (gMFI) upon 24 h of co-culture with three different tumor cell lines: U698M (**c**), A2058 (**d**) or Mel15 LCL (**e**). Tumor cells either expressed the mutated (mut mg) or wildtype (wt mg) minigene or were peptide-pulsed with KIF2C^{P13L} in increasing doses from 0.001 to 100 µM prior to co-culture setup. Mean and SD of three biological replicates are shown (E:T = 25,000:25,000).

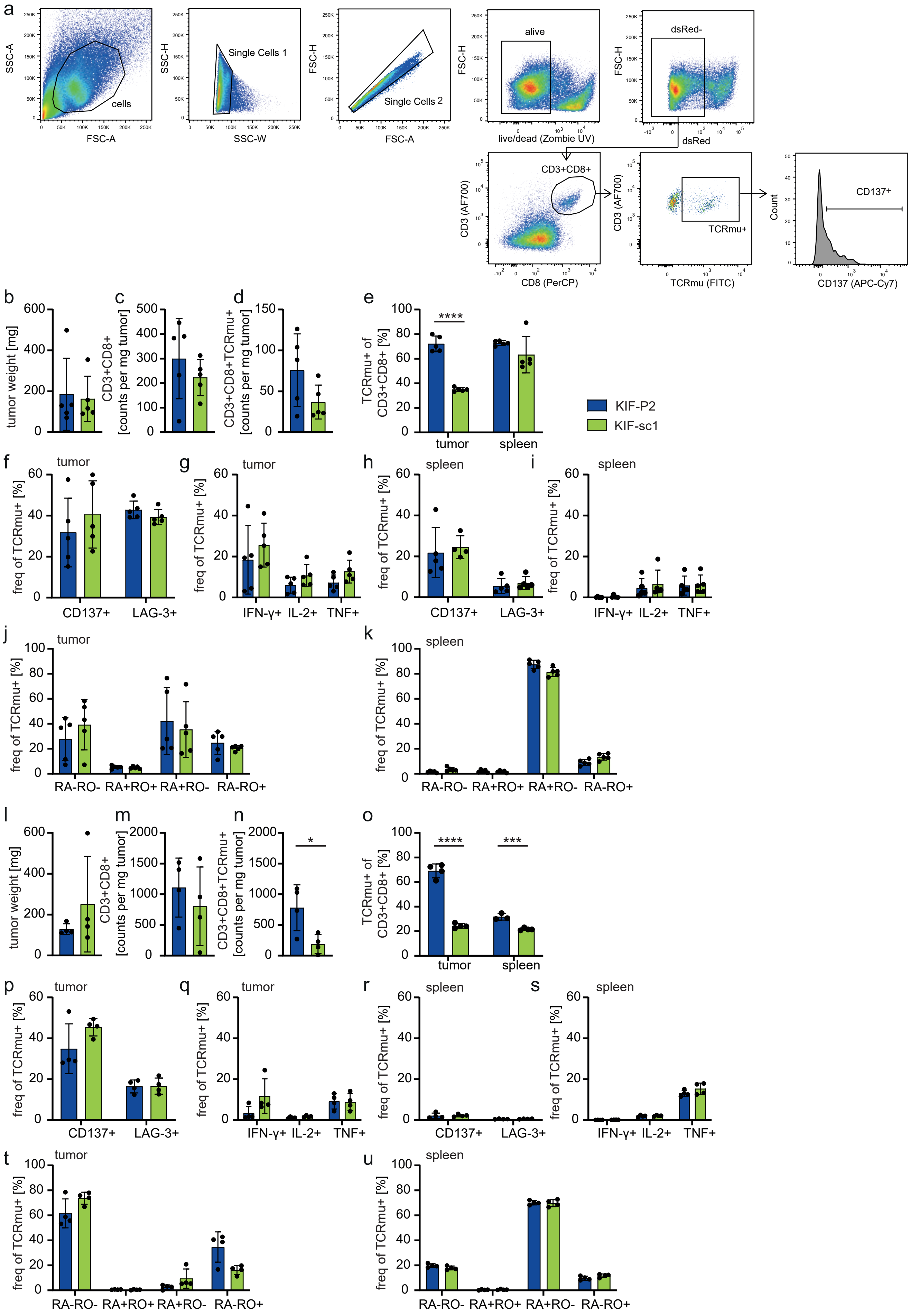

Supplementary Figure 10

Supplementary Figure 10. Tumor-infiltrating T cells upon first in vivo tumor encounter reveal TCRmu$^+$ T cell enrichment of KIF-P2 at the tumor site, but otherwise no functional or phenotypical differences between neoTCRs. a, Exemplary FACS gating strategy for ex vivo tumor analysis on day 5 after T cell injection upon first in vivo tumor encounter from left to right. Pseudocolor plots are depicted. **b-k**, Analysis of TILs on day 5 after T cell injection from two independent experiments in a lymphoma model (8x10^6 TCR-tg T cells per U698M-mut mg-tumor-bearing mouse; on day 0: 55% TCRmu$^+$ on T cells of donor B for **b-i** and 25% TCRmu$^+$ on T cells of donor A for **j, k**). **l-u**, Analysis of TILs on day 5 was repeated for a melanoma model (8x10^6 TCRmu$^+$ T cells of donor A injected on day 0 with 25% TCRmu$^+$ T cells). All analyses were performed in parallel to the lymphoma model (**b-k**). **b, l**, tumor weight of explants determined on day 5 ex vivo. **c**, **m**, FACS-based absolute count of CD3$^+$CD8$^+$ T cells per milligram (mg) tumor tissue, **d, n**, FACS-based absolute count of CD3$^+$CD8$^+$/TCRmu$^+$ T cells per mg tumor tissue. **e, o**, Frequency of TCRmu$^+$ of all CD3$^+$CD8$^+$ T cells detected at the tumor site or in the spleen on day 5. **f-i** and **p-s**, EC (activation markers CD137, LAG-3) and IC (cytokines IFN-γ, IL-2, TNF) FACS staining of TILs on day 5 comparing tumor (**f**, **g** and **p**, **q**) and spleen (**h, i** and **r, s**). **j, k**, In a second experiment with similar setup to the above mentioned analyses with comparable outcomes, FACS phenotyping of CD45RA (RA) and CD45RO (RO) was performed on TCRmu$^+$ TILs on day 5 for the tumor (**j**) and the spleen (**k**). **t, u**, Phenotyping CD45RA (RA) and CD45RO (RO) on day 5 was performed within the same experiment in the melanoma setting as in **l-s**. Statistical significance was calculated with unpaired t-test (*p≤0.05, ***p≤0.001, ****p≤0.0001). One dot represents one tumor-bearing mouse. Mean and SD are depicted.

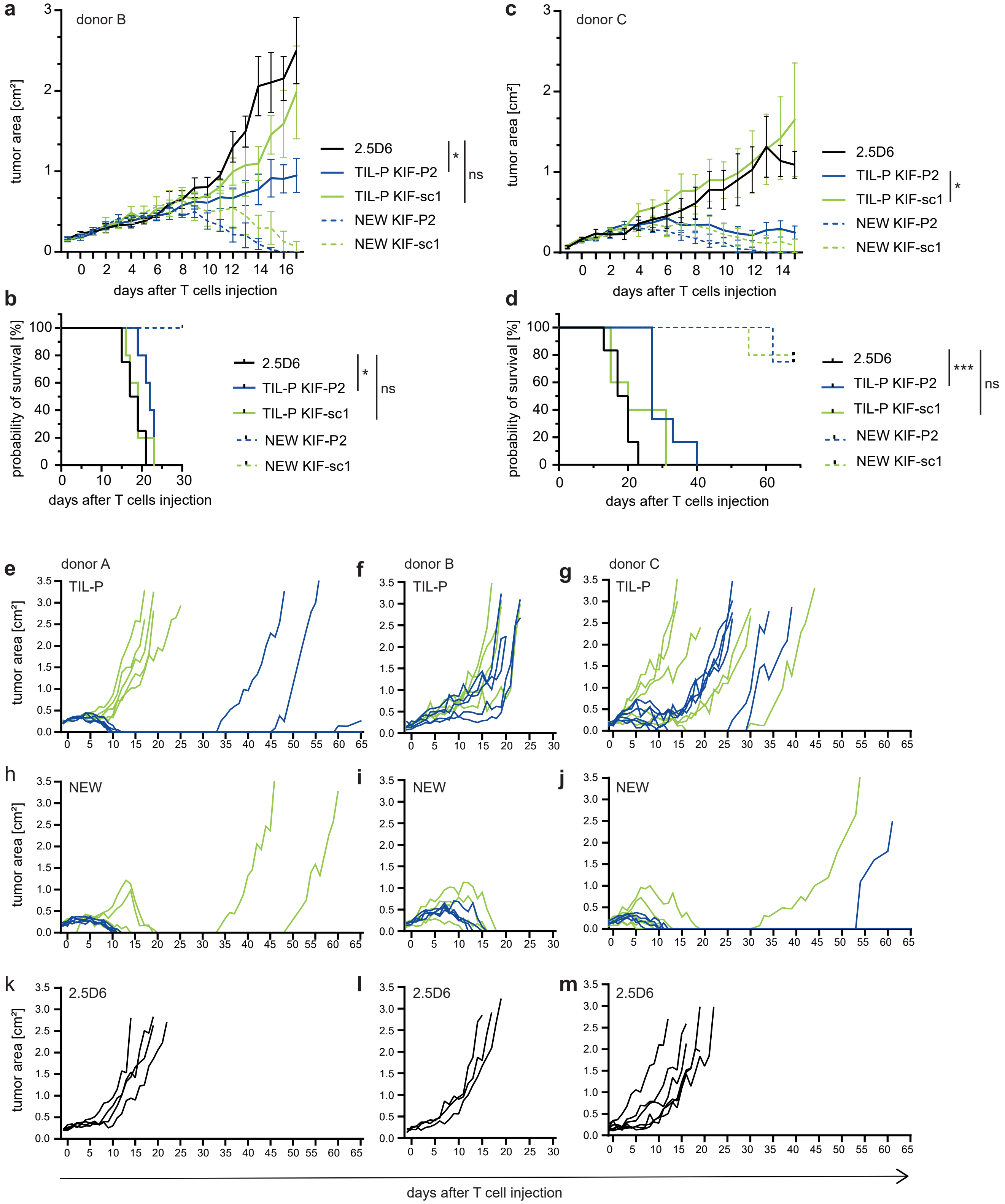

Supplementary Figure 11. TIL-P KIF-P2 exceeds tumor rejection potential of TIL-P KIF-sc1 upon in vivo rechallenge in T cell products from several healthy human donors. a-d, Accomplishing Figure **5b** and **c**, where the data of donor A is shown, the same experimental setting was performed with additional donors B (**a, b**) and C (**c, d**). **a, c**, Tumor growth kinetics of U698M-mut mg are displayed as tumor area (in cm^2) for NSG-mice until sacrifice of the first TIL-P mice after injection of in total 5x10^6 neoTCR-tg T cells (donor B: n=5 for TIL-P and n=4 for 2.5D6 and NEW; donor C: n=6 apart from n=5 for NEW KIF-P2 and TIL-P KIF-sc1). Mean and SEM describe tumor rejection. Statistical significance is calculated for the tumor area on day 17 (**a**) or day 15 (**c**) with one-way ANOVA and Tukey's multiple comparison test (*p≤0.05). **b, d**, Kaplan-Meier-survival curve is displayed for U698M-tumor-bearing mice injected with TCR-tg T cells (donor B: n= 5 for TIL-P or n=4 for 2.5D6 and NEW, donor C: n= 6 or n=5 for NEW KIF-P2 and TIL-P KIF-sc1). Statistical significance of survival of tumor-bearing mice was tested with Mantel-Cox test (*p≤0.05, ***p≤0.001). The growth curves and survival of NEW-conditions and 2.5D6 for donor B are also displayed in Figure **4d**, **e** for the first encounter setting and only serve as a reference here. **e-m**, Tumor growth curves for individual mice of each biological replicate (donors A, B and C) are displayed until the end of the experiment for TIL-P (**e-g**), NEW (**h-j**) and 2.5D6 (**k-m**) individually. KIF-P2 is depicted in blue, KIF-sc1 in green and 2.5D6 in black.

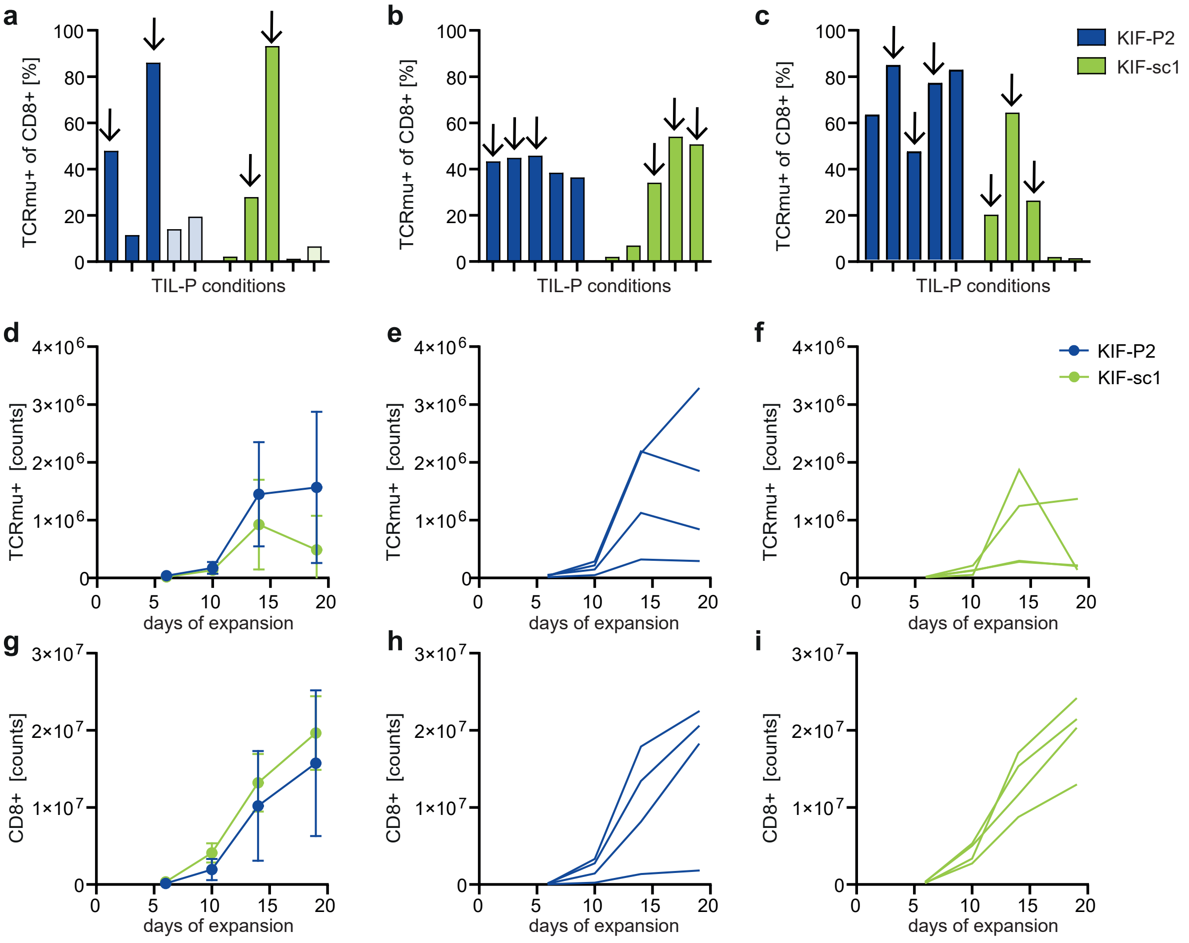

Supplementary Figure 12. KIF-P2 reaches more stable TCRmu surface expression and improved expansion during TIL-P production. a-c, Frequencies of TCRmu⁺ of all in vitro expanded CD8⁺ T cells from each individual mouse (each bar represents one initial tumor lysate from one mouse) on day 19 of TIL-P expansion for donors A (**a**), B (**b**) and C (**c**). Conditions combined per TCR for TIL-P for reinjection are marked (arrows). Transparent bars mark three conditions in the setup of donor A, where CD8⁺ T cells did not sufficiently expand and dsRed⁺ tumor represented >90% of cells on day 19. **d-i**, FACS-based absolute quantification of CD8⁺/TCRmu⁺ and CD8⁺ cells in TIL-P conditions in a separate experiment until day 19. Mean and SD (**d, g**) of single growth curves (**e, f** and **h, i**) are depicted.

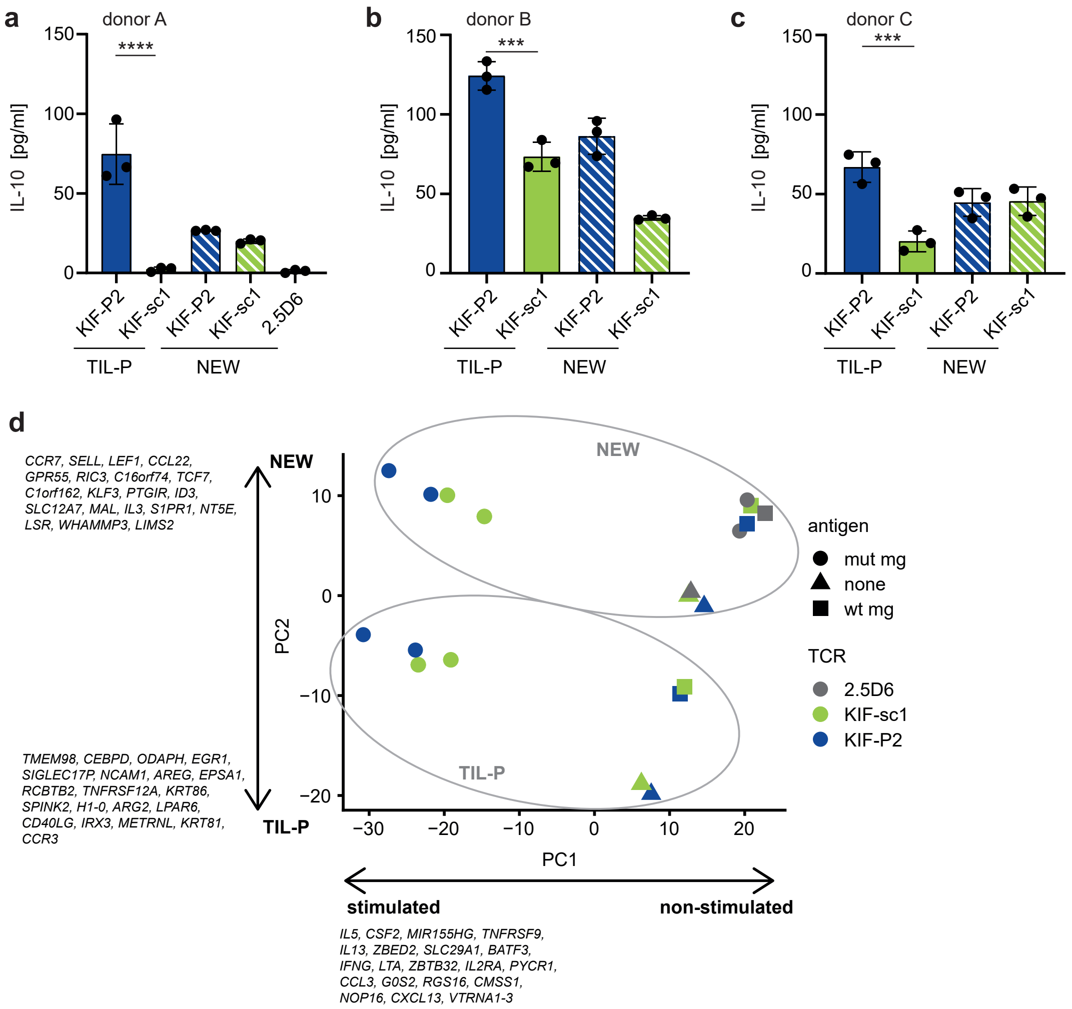

Supplementary Figure 13. Anti-inflammatory cytokine IL-10 is significantly upregulated in TIL-P KIF-P2 across human donors. a-c, Accompanying Figure **5d**, **e**, absolute values for secreted IL-10 are shown as measured by 13-plex legendplex analysis for in vitro co-cultures on the day of reinjection of TIL-P (day 21) for TIL-P and NEW cells from all three donors A (**a**), B (**b**) and C (**c**). Mean and SD of experimental triplicates (represented by dots) are depicted. Statistical significance is calculated with one-way ANOVA and Tukey's multiple comparison test (***p≤0.001, ****p≤0.0001). **d**, Principal component analysis (PCA) of bulk RNAseq data of restimulated CD8^{+} purified, in vitro restimulated TIL-P and NEW cells of donor B on day 21. PC1 (x-axis) distinguishes stimulated from non-stimulated conditions, while PC2 (y-axis) distinguishes NEW from TIL-P conditions; the top 20 genes responsible for the distinction are depicted on the ends of each axis.

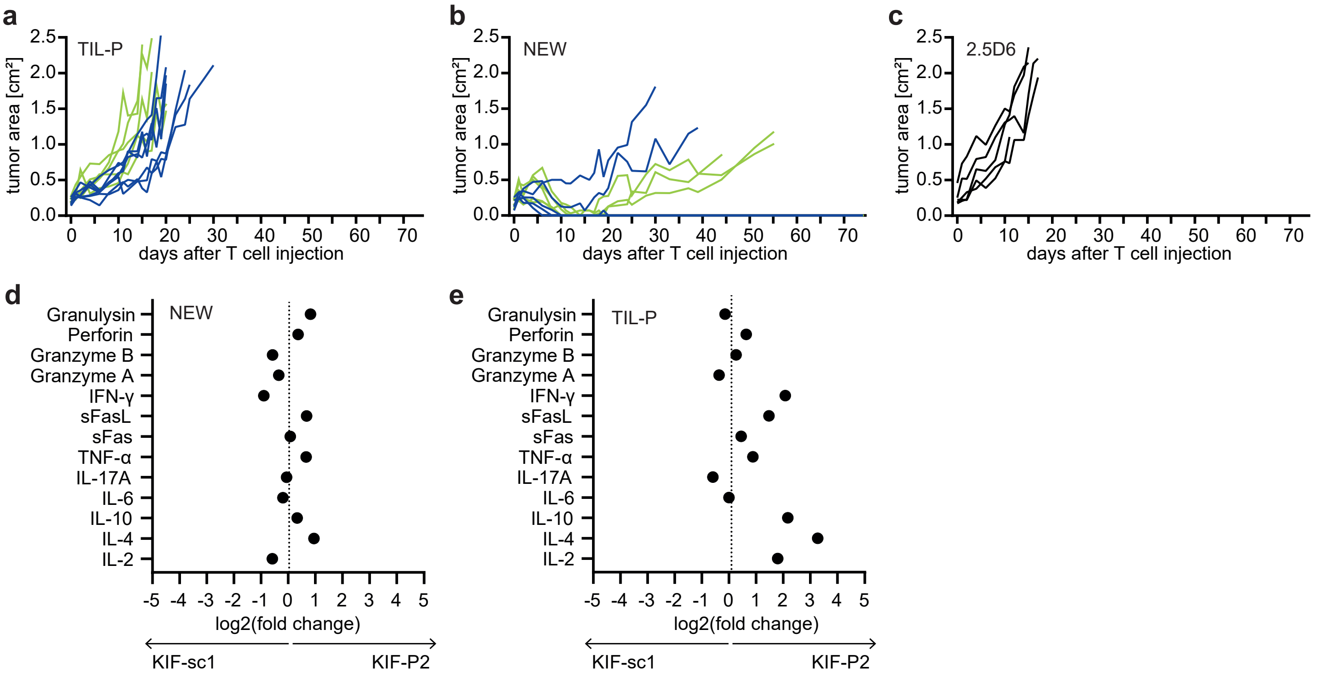

Supplementary Figure 14. Advantage in tumor killing upon rechallenge as well as IL-10 upregulation for TIL-P KIF-P2 can be detected independently of the entity. a-c, Tumor growth curves of each individual A2058-mut mg tumor-bearing mice are displayed until the end of the experiment for TIL-P (**a**), NEW (**b**) and 2.5D6 (**c**) individually. KIF-P2 is depicted in blue, KIF-sc1 in green and 2.5D6 in black. Individual growth curves of NEW conditions correspond to the data shown in Figure **4f**, **g**. **d-e**, Log2(fold change) depicted for the ratio KIF-P2:KIF-sc1 for cytokines secreted within 20 h of in vitro co-culture on the day of (re)injection of TIL-P or NEW T cell conditions (d0 of survival experiment) with A2058-mut mg. The comparison is drawn for NEW (**d**) and TIL-P (**e**) and the mean of three technical replicates of one human donor is depicted.

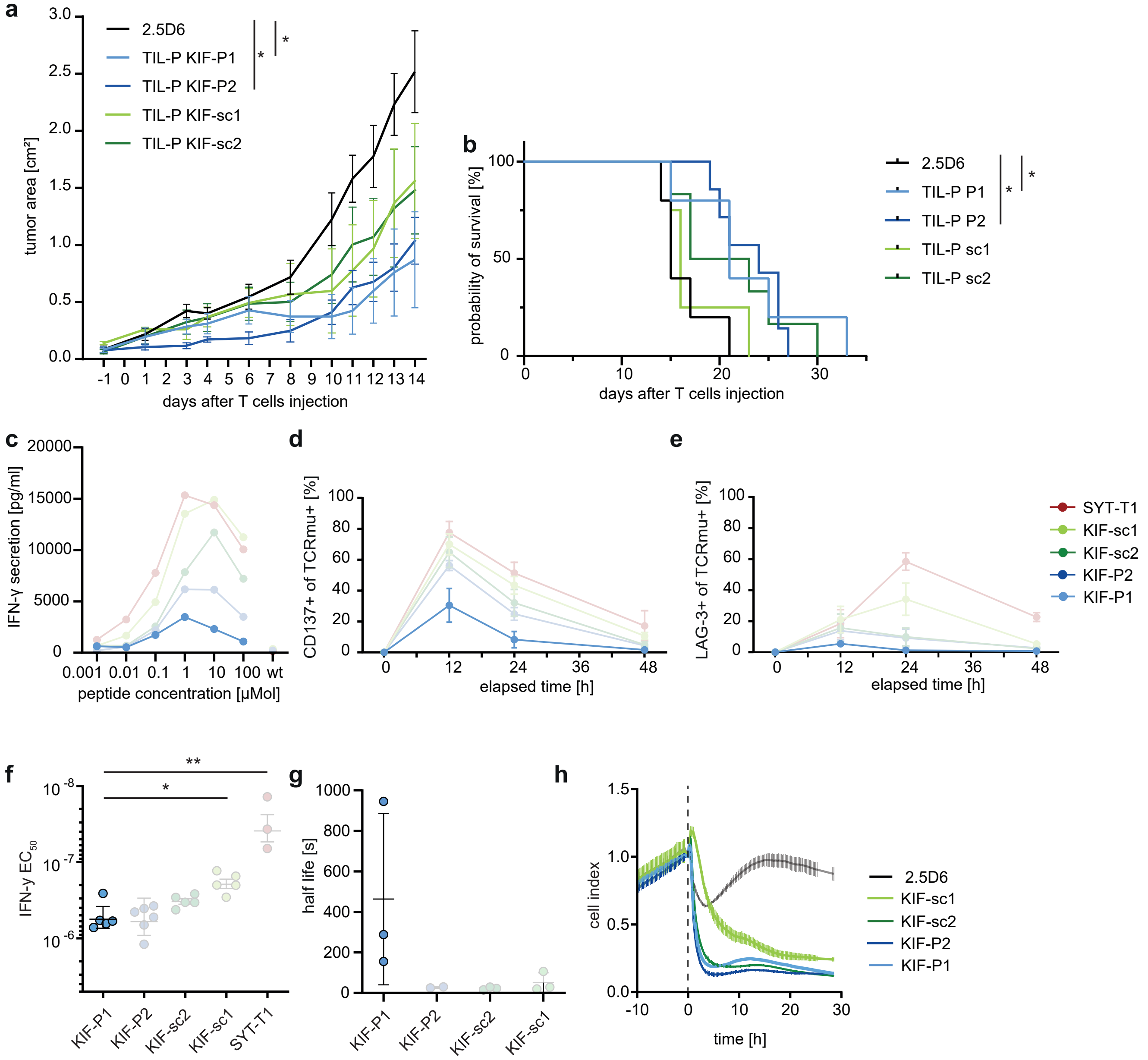

Supplementary Figure 15. TCR KIF-P1 shows strong in vivo killing capacity despite reduced surface expression. a, b, TIL-P rechallenge experiment was performed with all KIF2C^{P13L}-specific neoTCRs compared to 2.5D6 with a lowered effector cell number of $5x10^5$ TCR-tg T cells per mouse. NeoTCR KIF-P1 exhibited a markedly lower rate of TCRmu$^+$ during the first tumor encounter-sacrifice experiment (3% versus 25% TCRmu$^+$ for all other TCRs resulting in $1x10^6$ versus $8x10^6$ total TCRmu$^+$ T cells when administering the maximally possible number of T cells per mouse). Upon rechallenge equal number of $5x10^5$ TCRmu$^+$ per mouse administered; KIF-sc1: n=4; 2.5D6: n=5; KIF-P1: n=5; KIF-sc2: n=6¸KIF-P2: n=7. **a**, Tumor growth kinetics of U698M-mut mg are displayed as tumor area (in cm^2). Mean value and SEM for each group is depicted. Statistical significance is calculated for the tumor area on day 14 with one-way ANOVA and Tukey's multiple comparison test (*p≤0.05). **b**, Kaplan-Meier-survival curve is displayed for U698M-tumor-bearing mice injected with TCR-tg T cells. Survival was assessed with Mantel-Cox test (*p≤0.05, Mantel-Cox test). **c-e**, In vitro response of KIF-P1 within previously shown datasets of co-cultures (see Figure **3a**, **e**, **g** and legends for further explanation) irrespective of lower TCRmu$^+$ rate of KIF-P1 (2-3% versus equal dilution to 10% TCRmu$^+$ for all other TCRs). Direct comparability between KIF-P1 and the other TCRs is not given therefore the other neoTCRs are only shown (transparently) as reference. **f-g**, Functional (**f**) and structural avidity (**g**) measurement of KIF-P1 compared to the other neoTCRs (see also Figure **S5e-g**; previously shown data are shown transparently). Statistical significance is calculated with one-way ANOVA and Tukey's multiple comparison test (*p≤0.05, **p≤0.01). **h**, xCELLigence killing assay for melanoma cell lines A2058-mut mg of all sorted neoTCR-tg T cells compared to irrelevant TCR 2.5D6. T cells were added to previously cultured A2058 at 0 h and co-culture assessed for subsequent 30 h (E:T = 30,000:30,000).

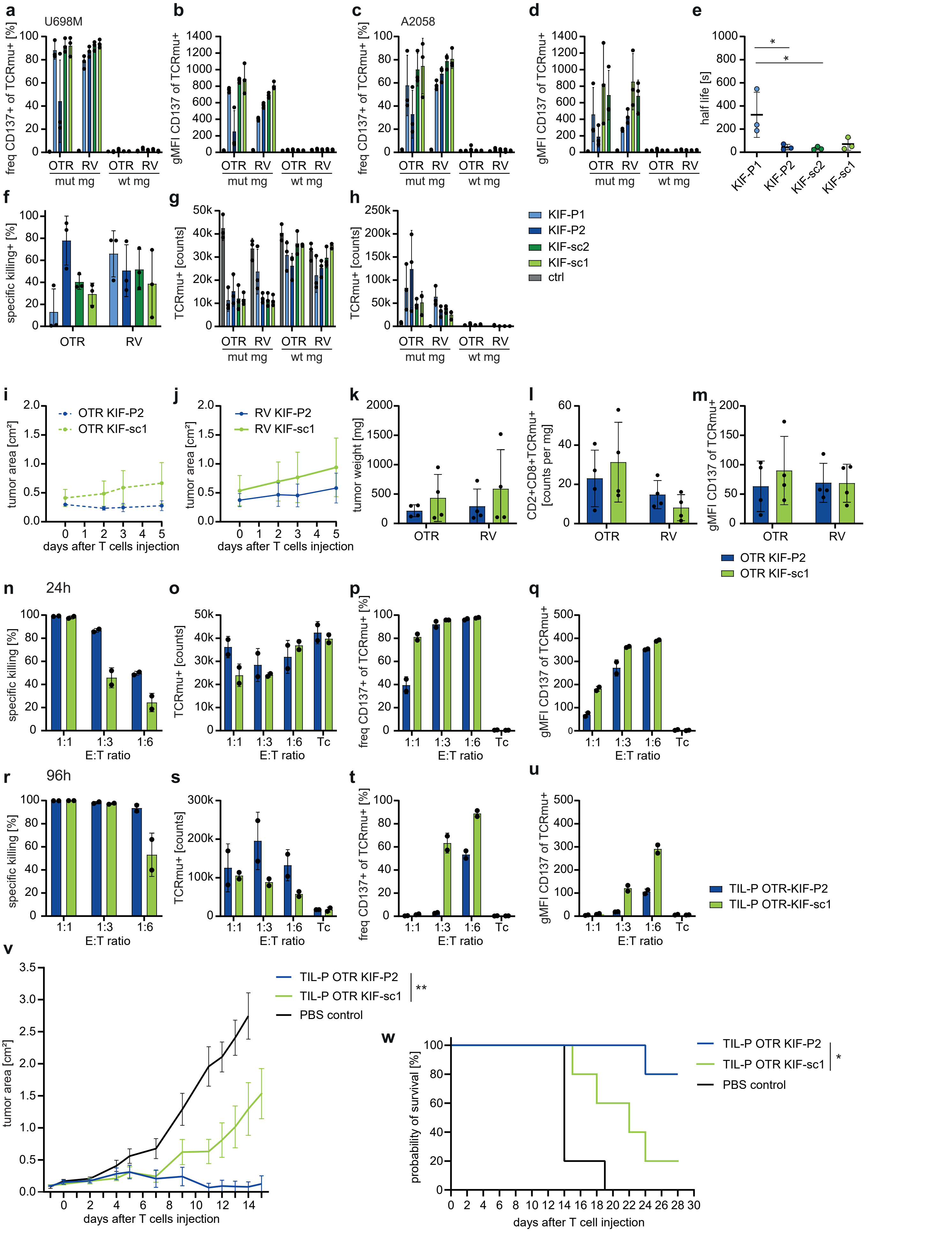

Supplementary Figure 16

Supplementary Figure 16. Functionality of orthotopically engineered TCR-T cells substantiate increased tumor control of KIF-P2 upon rechallenge. a-d, EC FACS staining of OTR versus RV TCR-engineered T cells upon co-culture with either U698M (**a, b**) or A2058 (**c, d**) for 20 h. Frequency of $CD137^+$ of all $CD2^+CD8^+/TCRmu^+$ (**a, c**) as well as gMFI of CD137 of $CD2^+CD8^+/TCRmu^+$ (**b, d**) cells are depicted. Three biological replicates are shown. **e**, FACS-based assessment of k_{off} rates using pMHC-multimers on OTR TCR-modified T cells. Data acquired as technical replicates from one biological donor. Statistical significance is calculated with one-way ANOVA and Tukey's multiple comparison test (*p≤0.05, **p≤0.01). **f-h,** FACS-based absolute quantification of residual $dsRed^+$ U698M tumor cells (**f**) or $TCRmu^+$ T cells (**g, h**) after 20 h (**f, g**) or 72 h (**h**) co-culture. Specific killing was calculated in relation to the remaining number of $dsRed^+$ tumor cells upon co-culture with an unspecific control TCR. **I-j**, Tumor growth kinetics of U698M-mut mg are displayed as tumor area (in cm^2) for NSG-mice until sacrifice on day 5 after injection of in total $8x10^6$ neoTCR-T cells engineered by OTR (**i**) or RV (**j**). TCR-T cells derive from one human donor (n=4 mice for each group). Mean and SEM are depicted. K, tumor weight of explants determined on day 5 ex vivo. **l**, FACS-based absolute count of $CD2^+CD8^+/TCRmu^+$ T cells per mg tumor tissue. **m**, EC FACS-based gMFI of CD137 of all $TCRmu^+$ T cells. **n-u**, FACS data of in vitro restimulated TIL-P-protocol expanded OTR-TCR-T cells comparing KIF-P2 with KIF-sc1 after 24 h (**n-q**) or 96 h (**r-u**) co-culture with U698M-mut-mg in different effector-to-target (E:T) ratios. Specific killing of $dsRed^+$ tumor cells was determined in comparison to tumor only (**n, r**). T cells (Tc) alone served as negative, unstimulated control. Remaining $CD2^+CD8^+/TCRmu^+$ cells (**o, s**) as well as frequency of $CD137^+$ (**p, t**) and level of CD137 of all $TCRmu^+$ T cells (**q, u**) were determined. Duplicates depict the TIL-P of those two mice from which TIL-P could be expanded for OTR-KIF-P2 or -sc1. **v**, Tumor growth kinetics of U698M-mut mg are displayed as tumor area (in cm^2) after injection of in total $5x10^6$ neoTCR-tg T cells (n=5 mice per group). TIL-P were pooled from both replicates per TCR-group shown in **n-u** each. 4 of 5 mice of the PBS control group achieved endpoint criteria already on day 14. Mean and SEM are depicted. Statistical significance is calculated for the tumor area of TIL-P OTR-KIF-P2 versus -sc1 on day 15 with two-tailed, unpaired t-test (**p≤0.01). **w**, Kaplan-Meier-survival curve is displayed for U698M-tumor-bearing mice injected with TCR-tg T cells (n=5 per group). Statistical significance of survival was tested on day 28 after T cell injection with Mantel-Cox test (*p≤0.05).

TCR	specificity	source	identification	first report	TCR frequency before enrichment	TCR frequency after enrichment
KIF-P1	KIF2C^{P13L}	PBMC	single-cell cloning and expansion	Bräunlein&Lupoli et al., [31]	133	3389
KIF-P2	KIF2C^{P13L}	PBMC	single-cell cloning and expansion	Bräunlein&Lupoli et al., [31]	20	629
KIF-sc1	KIF2C^{P13L}	PBMC	single-cell sequencing	newly identified	1	27
KIF-sc2	KIF2C^{P13L}	PBMC	single-cell sequencing	newly identified	0	23
SYT-P1	SYTL4^{S363F}	PBMC	single-cell cloning and expansion	Bräunlein&Lupoli et al., [31]	2	66
SYT-P2	SYTL4^{S363F}	PBMC	single-cell cloning and expansion	Bräunlein&Lupoli et al., [31]	1	14
SYT-T1	SYTL4^{S363F}	TIL	single-cell cloning and expansion	Bräunlein&Lupoli et al., [31]	0	8
SYT-T2	SYTL4^{S363F}	TIL	single-cell cloning and expansion	Bräunlein&Lupoli et al., [31]	0	15

Supplementary Table 1. NeoTCR clonotypes with specificity for KIF2C^{P13L} and SYTL4^{S363F} as well as TCR frequency before and after enrichment in the linked scTCR and transcriptome data set.

TCR	Recognition motif[a]	Number of antigens[b,c]
KIF-P1[d]	*X-X-X-L-X-L-A-I-K*	*60*
KIF-P2[d]	*X-X-X-L-X-L-X-I-K*	*> 400*
KIF-sc1	R-X-F-L-X-L-A-X-K	0
KIF-sc2	R-L-F-L-G-L-X-X-K	0
SYT-T1[d]	*X-R-I-A-F-F-X-X-X*	*6*
SYT-T2[d]	*X-R-I-A-F-F-X-X-X*	*6*
SYT-P1[d]	*X-R-I-A-F-F-X-X-X*	*6*
SYT-P2[d]	*X-R-I-A-F-F-X-X-X*	*6*

Supplementary Table 2. Recognition motif of neoTCRs extracted from alanine/threonine scan. Parts of this data have already been published [31] and were listed here next to the novel motifs of KIF-sc1 and -sc2 for direct comparison. (a) Recognition motifs are defined through T cell IFN-γ production in response to alanine/threonine scanned cognate epitopes, (b) Number of human proteins containing matching recognition motif according to ScanProSite, (c) Results derived from protein sequence database UniprotKB, Swiss-Prot (splice variants included), (d) Results have previously been published in [31] and are re-listed here to simplify direct comparison

	KIF-P1	KIF-P2	KIF-sc1	KIF-sc2	SYT-T1	SYT-T2	SYT-P1	SYT-P2
M_{Int}	56.1	11.5	0.7	1.5	4.1	7.9	15.3	2.9
M_{Int}-LN1	27.8	16.7	0.0	11.1	11.1	22.2	5.6	5.6
M_{Int}-LN2	33.3	16.7	0.0	5.6	11.1	16.7	16.7	0.0
M_{Lung}	36.6	46.6	9.5	1.4	2.3	1.0	1.4	1.1
M_{Lung}-LN	12.9	38.6	5.7	2.9	2.9	14.3	11.4	11.4

Supplementary Table 3. Percentage of each TCR contribution to the sum of all detected KIF2C^{P13L}- and SYTL4^{S363F}-specific T cells within M_{Int}, M_{Int}-LN1, M_{Int}-LN2, M_{Lung} and M_{Lung}-LN. Parts of this dataset were published before, i.e. for the previously identified TCRs KIF-P1, -P2, SYT-T1, -T2, -P1, -P2 [31] and are listed here for embedding the newly identified TCRs in the entire setting.

TCR	EC_{50} mean [nM]	EC_{50} SD [nM]	n (biological replicates)
KIF-P1[a]	558	177	5
KIF-P2[a]	602	307	6
KIF-sc1	194	61	5
KIF-sc2	329	60	5
SYT-P1[b]	45	28	3
SYT-P2[b]	163	136	3
SYT-T1[b]	39	26	3
SYT-T2[b]	70	55	3

Supplementary Table 4. EC_{50} values of neoTCRs as a measure for functional avidity. Data for TCRs SYT-T1, -T2, -P1, -P2, KIF-P1 and -P2 were pooled from previous experiments [31] and listed next to data retrieved for KIF-sc1 and -sc2 as depicted in Figure S4A. (a) Pooled results from previously published datasets [31] and novel experiments as depicted in Supplementary Figure S5. (b) Results have previous been published in [31] and are re-listed here to simplify direct comparison.
