# Supplementary material for: High-resolution profile of neoantigen-specific TCR activation links moderate stimulation to increased resilience of engineered TCR-T cells
Source: Nat Commun. 2024 Dec 3;15:10520. doi: 10.1038/s41467-024-53911-0 (PMC11615276; doi:10.1038/s41467-024-53911-0)
Supplement: Supplementary file 3 — Description of Additional Supplementary Files [file 41467_2024_53911_MOESM3_ESM.pdf]

Description of Additional Supplementary Files

Supplementary Data 1. Meta data of the single cell Seurat object, containing information about sample origin, number of features, cell state, clonotype, TCR and cluster distribution of each barcode.
