# Supplementary material for: High-resolution profile of neoantigen-specific TCR activation links moderate stimulation to increased resilience of engineered TCR-T cells
Source: Nat Commun. 2024 Dec 3;15:10520. doi: 10.1038/s41467-024-53911-0 (PMC11615276; doi:10.1038/s41467-024-53911-0)
Supplement: Supplementary file 5 — Reporting Summary [file 41467_2024_53911_MOESM5_ESM.pdf]

nature portfolio

Corresponding author(s): Angela Krackhardt

Last updated by author(s): Oct 22, 2024

Reporting Summary

Nature Portfolio wishes to improve the reproducibility of the work that we publish. This form provides structure for consistency and transparency in reporting. For further information on Nature Portfolio policies, see our Editorial Policies and the Editorial Policy Checklist.

Statistics

For all statistical analyses, confirm that the following items are present in the figure legend, table legend, main text, or Methods section.

n/a	Confirmed	
☐	☒	The exact sample size (*n*) for each experimental group/condition, given as a discrete number and unit of measurement
☐	☒	A statement on whether measurements were taken from distinct samples or whether the same sample was measured repeatedly
☐	☒	The statistical test(s) used AND whether they are one- or two-sided<br>*Only common tests should be described solely by name; describe more complex techniques in the Methods section.*
☒	☐	A description of all covariates tested
☐	☒	A description of any assumptions or corrections, such as tests of normality and adjustment for multiple comparisons
☐	☒	A full description of the statistical parameters including central tendency (e.g. means) or other basic estimates (e.g. regression coefficient) AND variation (e.g. standard deviation) or associated estimates of uncertainty (e.g. confidence intervals)
☐	☒	For null hypothesis testing, the test statistic (e.g. *F*, *t*, *r*) with confidence intervals, effect sizes, degrees of freedom and *P* value noted<br>*Give P values as exact values whenever suitable.*
☒	☐	For Bayesian analysis, information on the choice of priors and Markov chain Monte Carlo settings
☒	☐	For hierarchical and complex designs, identification of the appropriate level for tests and full reporting of outcomes
☒	☐	Estimates of effect sizes (e.g. Cohen's *d*, Pearson's *r*), indicating how they were calculated

Our web collection on statistics for biologists contains articles on many of the points above.

Software and code

Policy information about availability of computer code

Data collection	BD FACSDIVA (v.8.0.1), Glow-Max Discover (3.2.3), Immunospot (5.4.0.1), Illumina Sequence Analysis Software, QuantStudioTM 5 Real-Time-PCR-System (Applied Biosystems), xCELLigence® RTCA eSight-System, Dionex Ultimate 3000 RSLCnano system (Thermo Fisher Scientific) coupled to an Orbitrap Eclipse mass spectrometer (Thermo Fisher Scientific).
Data analysis	10x Genomics Cell Ranger (v4.0.0), Flow Jo (v10.8.1), GraphPad Prism (9.3.1), Immunospot (5.4.0.1), FragPipe software (version 21.1) with built-in search engine MSFragger version 4.0, IonQuant. R (v. 4.0.5) and Python (v. 3.8.12) with all required dependencies listed in the provided code for the analysis.<br>Scripts required for the transcriptomic analysis replication with the corresponding clonotype information are deposited upon the link to a Github repository: https://github.com/beltranLab/NeoTCR_Beltran_Krackhardt

For manuscripts utilizing custom algorithms or software that are central to the research but not yet described in published literature, software must be made available to editors and reviewers. We strongly encourage code deposition in a community repository (e.g. GitHub). See the Nature Portfolio guidelines for submitting code & software for further information.
